# Relationships between abiotic environment, plant functional traits, and animal body size at Mount Kilimanjaro, Tanzania

**DOI:** 10.1371/journal.pone.0174157

**Published:** 2017-03-20

**Authors:** David Schellenberger Costa, Alice Classen, Stefan Ferger, Maria Helbig-Bonitz, Marcell Peters, Katrin Böhning-Gaese, Ingolf Steffan-Dewenter, Michael Kleyer

**Affiliations:** 1 Department of Biology and Environmental Sciences, University of Oldenburg, Oldenburg, Germany; 2 Department of Animal Ecology and Tropical Biology, University of Würzburg, Würzburg, Germany; 3 Senckenberg Gesellschaft für Naturforschung, Biodiversity and Climate Research Center, Frankfurt, Germany; 4 Institute of Experimental Ecology, University of Ulm, Ulm, Germany; 5 Institute for Ecology, Evolution & Diversity, Goethe University Frankfurt, Frankfurt (Main), Germany; University of Saskatchewan, CANADA

## Abstract

The effect-response framework states that plant functional traits link the abiotic environment to ecosystem functioning. One ecosystem property is the body size of the animals living in the system, which is assumed to depend on temperature or resource availability, among others. For primary consumers, resource availability may directly be related to plant traits, while for secondary consumers the relationship is indirect. We used plant traits to describe resource availability along an elevational gradient on Mount Kilimanjaro, Tanzania. Using structural equation models, we determined the response of plant traits to changes in precipitation, temperature and disturbance with and assessed whether abiotic conditions or community-weighted means of plant traits are stronger predictors of the mean size of bees, moths, frugivorous birds, and insectivorous birds. Traits indicating tissue density and nutrient content strongly responded to variations in precipitation, temperature and disturbance. They had direct effects on pollination and fruit traits. However, the average body sizes of the animal groups considered could only be explained by temperature and habitat structure, not by plant traits. Our results demonstrate a strong link between traits and the abiotic environment, but suggest that temperature is the most relevant predictor of mean animal body size. Community-weighted means of plant traits and body sizes appear unsuitable to capture the complexity of plant-animal interactions.

## Introduction

Plants are the primary producers of terrestrial ecosystems. A large body of literature in plant ecology has emerged around plant functional traits, i.e. plant properties indicating a functional relationship with the abiotic environment or with biotic components of ecosystems [1–2]. The notion of the translation of changes in the abiotic environment to ecosystem functioning mediated by plant functional traits is described by the effect-response framework [3]. It assumes traits respond to changes in the abiotic environment and affect ecosystem functioning, including animal community composition. Many studies investigated the simultaneous role of plant response and effect traits in relation to properties of the vegetation compartment of ecosystems, such as standing biomass, productivity, and litter decomposition [4–7]. Rarely, however, have studies addressed the links between abiotic environmental conditions, plant functional traits and other trophic levels. Recently, Lavorel et al. [8] proposed an extension of the effect-response framework, employing a multitrophic perspective.

Body size is one of the most prominent characteristics of animal species. It has implications for the life history and the role of species within ecosystems [9]. The relationships of body size to metabolic rate, growth, mortality, locomotion, and nutrition, among others, have led to many hypotheses concerning the variability and range of body sizes within organism groups in relation to energetic constraints, textural discontinuity of habitats, biotic interactions such as predation and competition, and biogeographical and phylogenetic limitations [10–11].

On the level of individual species, observations on closely related extant and fossil taxa have led to the formulation of several rules, the best-known of which is Bergmann's rule [12]. It states that among closely related species, taxa in colder climates tend to be larger than those from warmer regions. The reason for this pattern is seen in the relatively lower heat loss of larger animals compared to smaller ones. Previous work discussed Bergmann's rule for endo- and ectothermic animals (see [13] for an overview). For the latter, higher frost resistance, larger energy reserves for surviving starvation periods, and longer growth and development time at low temperatures have been brought forward to explain negative relationships of temperature and size [14–16]. A more general explanation can be derived from the notion that specific metabolic rate, i.e. metabolic rate divided by body mass, is lower for large animals compared to small ones, resulting in a more efficient use of energy [17].

Because of much discordance with Bergmann's rule [18–20], the assumption of temperature being the main driver of animal body size has been challenged and resource availability was proposed as another important determinant of body size [19, 21–22]. Resource availability depends on primary productivity; directly for herbivores [23], and indirectly for predatory animals [24–26].

Here, we present a novel application of the effect-response framework using plant functional traits to predict variation in the body size of animal taxa along an elevation gradient at Mount Kilimanjaro, Tanzania. We ask how plant traits respond to variations in precipitation, temperature and disturbance by land use and how they affect mean animal body size. Functional traits were selected to express resource availability for moths, bees, frugivorous birds, and insectivorous birds. Moths rely on plant leaves during growth and development, and nectar at the reproductive stage. Bees are entirely dependent on pollen and nectar. Frugivorous birds feed on fleshy fruits. Insectivorous birds only indirectly depend on plant functional traits, as they consume insects, which rely on plants for nutrition. We hypothesized that a larger supply of leaves, nectar, fruits, and insects should result in an increase in the body size of moths, bees, and birds, respectively. Leaf biomass was approximated by the total plant biomass of a community, nectar quantity by the proportion of insect-pollinated plants. Supply of fruits was recorded in the field and insect availability for birds was approximated by the cumulative body size of bees and moths in each plot. Herbivores often prefer leaves with a low C/N ratio [27]. To account for this, we included a variable aggregating leaf C/N ratio and toughness in the analysis of moth body size, expecting lower C/N ratios to be associated with larger body size. The effect of these factors on the community-weighted mean body size of moths, bees, frugivorous birds, and insectivorous birds was investigated for each group separately.

Of the multiple factors possibly affecting body size distributions, temperature as a proxy for energetic constraints and habitat structure were additionally considered. Habitat structure was described by total plant biomass. For moths, this factor expressed both resources and habitat texture, corresponding to differences between open areas and forests. For bees and moths, the more patchy distribution of resources expected in forests should lead to increases in body size [28]. For birds, on the contrary, multilayer canopies are an obstacle to mobility and may favor smaller species [29].

Structural equation modeling was applied to investigate the complex relationships between plant functional traits, total plant biomass, the abiotic environment, and the body size of the different animal groups. This implied additional hypotheses concerning the relationships of plant functional traits and the abiotic environment (Table 1). Total plant biomass and leaf economics were expected to increase with precipitation and decrease or increase with disturbance, respectively [30–31]. Leaf economics summarizes several correlated plant functional traits describing a plant strategy gradient from fast growth and nutrient turnover to persistence and nutrient retention [32]. Fast growth and nutrient turnover are essentially linked to high resource supply and disturbance frequencies. Total plant biomass should positively affect nectar and fruit availability, while disturbance should be related to high nectar, but low fruit availability. We made use of a unique dataset including climatic data, plant traits, plant biomass, and animal abundance data sampled synchronously on sixty plots at Mount Kilimanjaro. Our study sites covered a wide range of tropical habitats varying strongly in mean temperature, precipitation, and human disturbance and are therefore ideal for testing theories about the influence of both temperature plant functional traits on consumer community traits.

**Table 1:** Hypotheses defining the structural equation models. The structural equation models relating plant traits to animal body size CWMs were implemented according to expected relationships between abiotic environment, plant functional traits, animal body size CWMs, and animal cumulative body size.

Model	Response	Predictor	Hypothesized Relationship	Explanation
M, B, F, I	Disturbance	Temperature	+	Anthropogenic activities are strongest at low elevations close to settlements
M, B, F, I	Body size CWM	Temperature	-	More efficient energy use of larger animals in cold environments [12, 17]
I	Cumulative body size*	Temperature	-	More effective energy use of larger animals in cold environments [12, 17]
M, B, F, I	Leaf economics	Precipitation	+	Tougher leaves conserve water [32]
M, B, F, I	Total plant biomass	Precipitation	+	Water supply limits primary productivity [30]
M, B, F, I	Leaf economics	Disturbance	+	Fast growth and turnover necessary [33]
M, B, F, I	Total plant biomass	Disturbance	-	Biomass removal
B	Body size CWM	Disturbance	+	Large species can better exploit fragmented habitats [34–35]
M, B	Insect-pollinated plants CWM	Leaf economics	+	High-leaf economics plants in study area are mostly insect-pollinated weeds
F	Bird-dispersed fruits CWM	Leaf economics	-	High-leaf economics plants mostly produce small wind-dispersed seeds
M	Body size CWM	Leaf economics	+	Lower C:N ratio, higher food quality [36]
M, B	Proportion of insect-pollinated plants	Total plant biomass	+	Reduced wind speed through persistent foliage in forests makes wind-pollination less effective [37]
F	Bird-dispersed fruit CWM	Total plant biomass	+	Advantage of longer transport distances of large seeds through animals in forests [38]
M, B, F, I	Body size CWM	Total plant biomass	+/-	Increase in resources for moths [19, 23], longer foraging routes favor large insects, for birds, smaller species can better travel in canopies [10]
M, B	Cumulative body size*	Total plant biomass	+	Higher primary productivity resulting in larger animal biomass [39–40]
M, B	Body size CWM	Proportion of insect-pollinated plants	+	Increase in resources through more biomass [19, 23]
F	Body size CWM	Bird-dispersed fruit CWM	+	Increase in resources through more biomass [19, 23]
I	Body size CWM	Cumulative body size*	+	Increase in resources through more biomass [19, 23]

Abbreviations: M: moth model, B: bee model, F: frugivorous bird model, I: insectivorous bird model.

*Moths and bees.

## Materials and methods

### Study region

Mount Kilimanjaro was selected as the study system. It is located in Northern Tanzania at 3.1°S 37.4°E. It is the highest free-standing mountain in the world and covers an area of approx. 4000 km^2^. The elevation gradient ranges from the lowlands at 800 m a.s.l. to the peak at 5892 m a.s.l. Precipitation values range from 550 mm•a^-1^ to 3600 mm•a^-1^, with the highest amount of rainfall occurring at mid-elevations in the forest belt and the lowest amounts in the alpine zone and the plains surrounding the mountain [41]. Anthropogenic disturbance is expressed differently upon the mountain. Fires, occasional timber extraction, and small-scale collection of other forest products affect higher elevations, while agriculture involving fertilizers and herbicide application is practiced in lowland areas.

### Data collection

All necessary permits were granted from the Tanzanian Commission for Science and Technology, the Tanzanian Wildlife Authority, and Tanzania National Parks (340-ER-NA-96-44, TNO/HQ/C.10/13/VOL.III).

Collection of plant functional traits and data on animal taxa took place on 60 plots, each of 0.25 ha, in the twelve major vegetation types at the mountain between August 2010 and November 2012 (S1 Table). Plots were distributed equally among vegetation types, five plots belonging to each type.

Undisturbed plots at low elevations around 1000 m a.s.l. were dominated by annual grasses and drought-tolerant trees, whereas weeds characterize cultivated areas. At 1600 m, coffee plantations and agroforestry systems harbored mostly dicotyledonous weeds. Grasslands were dominated by several taxa of Poaceae. Woody life forms accounted for the largest part of plant biomass from middle to upper elevations, with a transition from rainforest to cloud forest characterized by small trees and high lichen abundance at around 3000 m. Alpine vegetation occurred up to 4500 m and was mainly composed of shrubs, perennial herbs, and grasses.

Mean temperature was derived from several years of continuous measurements with automatic data loggers covering the time period of our data collection [42]. Annual precipitation data was derived from the Kilimanjaro rainfall model [43]. Disturbance was calculated as a composite metric including the effects of land use at local and landscape scales ([44], S1 File).

Plant functional traits were collected from the most abundant vascular plant species, accounting for 80% of total plant biomass on each plot. Abundance was defined as percent cover determined from vegetation surveys. Cover was estimated for each stratum depending on the vegetation structure of the plot. Cover of all strata was summed up for each species and standardized to a plot total of 100%. Plant functional traits were chosen to indicate vegetative growth, persistence, and reproductive characteristics. Fifteen individuals per species were sampled from different plots within the elevational distribution range for specific leaf area (SLA), leaf dry matter content (LDMC), stem specific density (SSD), leaf nitrogen content (leaf N_mass_), leaf phosphorus content (leaf P_mass_), fruiting frequency, fruit number, and fruit size. Additionally, the total plant biomass per plot was calculated with allometric equations using complete tree and undergrowth inventories (details in [45]). Sampling and processing of plant material followed Kleyer et al. [46].

Percentage cover values from vegetation surveys were used to calculate community-weighted mean trait values for each plot (CWM, [47]). To describe leaf palatability for moths, the first axis of a principal components analysis using SLA, LDMC, SSD, leaf N_mass_ and leaf P_mass_ was extracted and termed "leaf economics" (for correlations of variables see S2 Table). The traits used are related to the worldwide leaf economics spectrum [32, 48], reflecting a gradient from plants with fast resource use and nutrient turnover to those with slow and persistent growth. The former are characterized by soft tissues with high nitrogen content and are generally preferred by herbivores, due to their easier digestion and higher nutritional value [32]. All data were collected on the same plots within a common time frame to avoid confounding effects of spatial and temporal variability.

To quantify nectar and fruit availability on the plots, pollination and dispersal syndromes were extracted from the Flora of Tropical East Africa [49]. Nectar availability is difficult to quantify, as nectar amounts per flower, flowering times, and the proportion of tissue invested in flowers are highly variable between species. This resource was approximated by the abundance-weighted proportion of insect-pollinated plant species. To assess food resources for frugivorous birds, average fruit numbers per individual plant sampled in the field (fn), average fruit size (fs), fruiting frequency (ff), and relative abundance (ra) were used to calculate the bird-dispersed fruit CWM (bdc) according to the following formula:

$$bdc=\sum ra•mean\left(fn\right)•mean\left(fs\right)•ff•\delta_{bdc}$$

The sum is taken over all plant species in the respective plot. δ_bdc_ equals 1 for species producing fruits consumed by birds and 0 otherwise. Birds were observed through point counts, both in the dry and wet season to include temporal variation (see [50] for methodology). Bees were sampled with pan traps on the forest floor and in the canopy of woody vegetation. Sampling was repeated several times to account for temporal variation (see [44]). Moths were caught with an automatic light trap with a superactinic light tube (6 watt, Fritz Weber Entomologiebedarf, Stuttgart, Germany) as light source. This was repeated in dry and wet seasons. Body mass of birds was derived from Dunning Jr [51] and used as a proxy for body size [52]. Within animal groups of similar body structure, body length and size are highly correlated [53]. For bees, body size was approximated by the highly correlated intertegular distance (ITD, [54]). Moth body length was measured using a binocular Leica stereomicroscope with a calibrated ocular micrometer.

As with plant traits, animal body size was weighted by species abundance, yielding a body size CWM for each taxon or guild, to avoid giving rare species the same weighting as abundant ones [21]. For simplicity, in the following community-weighted mean body size is referred to as body size. Cumulative body sizes of moths and bees as food resources for insectivorous birds were calculated as the sum of the individual body sizes of all individuals of the respective groups sampled. For plot means and ranges of plant functional traits, animal body size, cumulative bee ITD, and cumulative moth body length see S3 Table. Analyses were also performed with community means not weighted by abundance, but as there were no substantial differences, only CWM results are presented.

### Data analysis

Structural equation modeling with mixed effect models was applied to relate mean body size to environmental data and plant functional traits [55]. We used the directional-separation-test (d-sep, [56]) to confirm or reject the assumed relationships between variables in the structural equation model. d-sep works by testing individual regressions between all pairs of variables of the model. Direct relationships, i.e. variables connected by arrows, should turn out to be significant. Indirect relationships, i.e. variables connected by several arrows through other variables, should not be significant. However, for indirect relationships, not the variables themselves, but their residuals from regressions on their direct connections, are tested. This approach delivers P-values for the individual relationships between variables and an overall model fit. We applied mixed effect models for regression, because they offer the possibility of including plot-specific and vegetation type-specific random effects [55]. We assumed linear relationships between all parameters within the limited ranges of the variables investigated. Variables were standardized prior to analysis. To obtain correct P-values, we followed the recommendations of Barr et al. [57] and included random slopes and random intercepts. All calculations were done in R [58]. For mixed effect models, the lmer function in R package lme4 was used [59] with Gaussian error structure obtaining maximum likelihood estimates (option REML = F). Separate models were run for each animal group to keep total variable numbers low with regard to the number of observations. For each group, the hypothesized model including all predicted interdependencies was tested first. Then, an improved model omitting the non-significant terms at a P-value of 0.05 was run. Coefficients, P-values and R^2^ values for both initial and "significant" models are reported. Table 1 lists the detailed hypotheses defining the structural equation models.

## Results

**Fig 1:**
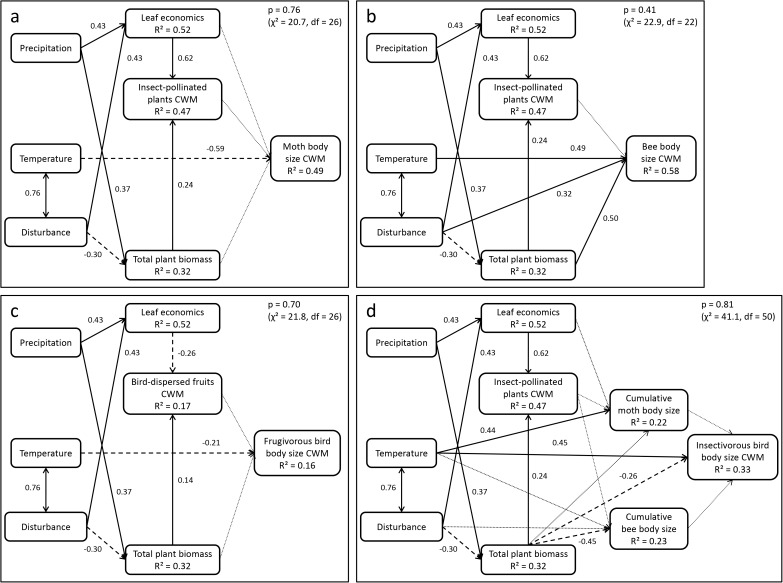
Structural equation model showing the relationship between the abiotic environment, disturbance, plant functional traits and community-weighted means of animal body size for moths, bees, insectivorous and frugivorous birds. Standardized coefficients are given for positive (solid lines) and negative (dashed lines) relationships. Double-headed arrows denote correlations of predictors. Dotted lines indicate hypothesized relationships that were not supported by the data, i.e. which turned out to be non-significant in the regression models. R^2^ values are marginal, i.e. give fixed effect explained variance. See S4 Table for unstandardized coefficients, individual P-values, and conditional R^2^ values. (a) Moths, (b) Bees, (c) Frugivorous birds, (d) Insectivorous birds

There were large differences in the individual relationships and effect sizes inferred from the structural equation models (Fig 1, see S4 Table unstandardized coefficients, individual P-values, and conditional R^2^ values). Overall, expectations concerning the abiotic environment and plant functional traits were confirmed by the analyses, but relationships between plant functional traits and body size were much weaker and not always according to the hypotheses. For individual variables, explained marginal R^2^, i.e. the proportion of variance explained by linear regressions, was between 0.16 and 0.58, while conditional R^2^ values expressing the summed effect of linear regressions and accounting for vegetation type-differences ranged from 0.16 to 0.89 (S4 Table). Overall model probability values ranged from 0.41 to 0.81. Exclusion of non-significant terms from the initial hypotheses (dotted lines in Fig 1, S4 Table) had a positive effect on model R^2^ values, especially for birds.

Relationships between the abiotic environment and plant traits and biomass were the same for all models. Leaf economics and total plant biomass were positively related to precipitation. Disturbance had a negative effect on total plant biomass, but a positive one on leaf economics. The proportion of insect-pollinated plants was explained by total plant biomass and leaf economics, both with positive coefficients. Bird-dispersed fruit CWM was negatively related to leaf economics and positively to total plant biomass.

Moth and frugivorous bird body size were explained by temperature alone, while body sizes of bees and insectivorous birds were also related to total plant biomass. Both moth and frugivorous bird body size decreased with increasing temperature (Fig 1A and 1C). As hypothesized, bee body size increased with disturbance, but decreased with temperature (Fig 1B). Contrary to our expectation, insectivorous bird body size was positively related to temperature (Fig 1D). Total plant biomass affected bee body size positively, but was negatively related to insectivorous bird body size, in line with our hypotheses. Resource availability had no positive effect on insectivorous bird body size, as indicated by the lack of a significant relationship with cumulative body size of moths and bees. Cumulative body size of moths in turn was positively related to temperature, while cumulative body size of bees decreased with increasing total plant biomass.

## Discussion

In our study, we modeled the response of plant functional traits to environmental gradients and their effect on body sizes of different animal taxa. We assumed that body sizes depend directly on temperature and disturbance and indirectly on plant traits and total plant biomass as indicators of resource availability or habitat structure. Temperature affected the body size of all animal groups considered and total plant biomass affected body sizes of bees and insectivorous birds. Plant traits were unrelated to the body size of any of the groups, suggesting that either resource availability may not be the main driver of body size, or community-weighted means of plant traits did not reflect resource availability adequately.

### Environment – plant functional traits

Precipitation and disturbance were linked to increases in leaf economics. This variable aggregated a suite of correlated traits such as specific leaf area, leaf nitrogen and phosphorus content, leaf dry matter content, and stem specific density. It indicates a gradient ranging from relatively slow growth and conservation of resources to fast growth and rapid turnover of resources in leaves termed the leaf economics spectrum [32]. High values of leaf economics occurred in fast-growing plants with highly palatable tissues, while low values were found in slow-growing plants investing in long-lasting structural components. Whether traits indicating the leaf economics spectrum are linked to precipitation on global scales is still equivocal [60]. At Mount Kilimanjaro, precipitation was the strongest driver of total plant biomass and leaf economics, whereas temperature had almost no effect, in contrast to findings on a global scale [60]. The strong positive effect of disturbance (logging, grazing) on leaf economics is concordant with other studies worldwide (e.g. [31, 61]).

The positive and negative relationships between leaf economics and the proportion of insect-pollinated plants and bird-dispersed fruit CWM, respectively, were expected due to the functional differences between undisturbed savanna and forests on the one hand and agricultural areas on the other. At Mount Kilimanjaro, grazed and ploughed habitats mostly harbor fast-growing dicotyledonous weeds with rather high leaf economics values. These weeds are pollinated by insects, but produce mostly wind-dispersed seeds, instead of bird-dispersed fruits. Seed dispersal by animals is often strongly affected by disturbance, but pollination is not [44, 62]. Large differences between marginal and conditional R^2^ values were evident in total plant biomass, in the proportion of insect-pollinated plants, and in bird-dispersed fruit CWM. This could be caused by non-linear relationships of predictors and traits or factors not accounted for by the predictors [63].

### Environment – animal body size

Bergmann's rule and its recent formulations in the framework of the metabolic theory of ecology [17] predict decreases in body size with increasing temperature. Moths and frugivorous birds were in accord with this expectation, in line with previous studies (e.g. [14, 20, 64]). Conversely, insectivorous bird size increased with temperature, contrary to most other studies on birds [65]. Unlike patterns of body size along elevational gradients in the Alps [66], mean body size of bees at Kilimanjaro did not follow Bergmann's rule. Bees of smallest body size occurred in the alpine *Helichrysum* shrubland situated at highest elevations, suggesting increased extinction probabilities for large-bodied bee species in low-energy habitats [67]. The only other variable found to increase bee body size was disturbance, probably because most wild bees that visit crops or ruderal plants in disturbed habitats nest in natural habitats [34] and isolated resources in disturbed habitats can better be exploited by large-bodied bees species with larger foraging ranges [35]. However, for social species, which accounted for a small proportion of bee species richness, but for a considerable part of the specimens, Kaspari and Vargo [68] noted that colony size rather than individual body size should follow Bergmann's rule. Nevertheless, this could not be tested, as colony size data were not available.

### Plant functional traits – animal body size

The food availability hypothesis [21] and the textural discontinuity hypothesis [10] justified the expectation of a strong influence of plant functional traits and total plant biomass on animal body size. However, we did not find any significant relationship between body size and CWMs of functional traits that indicate resource availability. Assuming the "resource availability hypothesis" were correct, a shortcoming of the approach presented here might be that the traits used were not precise enough indicators of the "true" food resources. We used leaf and stem traits, that indicated a gradient from carbon-rich, hard tissue to soft, nutrient-rich tissue as an indicator for moth food availability [69]. This, however, may not adequately reflect the amount of palatable tissue for moths, as Lepidoptera tend to be specialized to particular host plants [70]. In moths, a correlation between body size distributions and size distributions of floral resources has been observed [71], indicating that nectar quantity may have predictive potential for body size. Still, the CWM of insect-pollinated plants may be a too coarse estimate of the nectar actually available for bees and moths, as flower rewards, inflorescence sizes and nectar contents can vary strongly across plant species [72].

Total plant biomass had a significant effect on the body size of bees. Rainforests were characterized by the highest structural complexity, indicating that larger distances to food sources select for larger bee species [28, 44]. Moths however, did not respond in the same way, possibly because the caterpillar life stage exerts a strong influence on body size and leaves are a rather abundant resource, which do not force caterpillars to forage long distances in both open landscapes and forests.

Insectivorous bird body size was not significantly related to resource availability, probably because the latter was approximated by the cumulative body sizes of all moth and bee individuals per plot. These measures should be good proxies of overall insect abundance in most vegetation types, but probably not at high elevations. Bee abundance in the alpine zone was high, but other insects were rather rare (pers. obs.). For leaf herbivores, this was evidenced by the virtual absence of damaged foliage at high elevations. Including other insect taxa in our study would likely change our estimate of the food availability pattern for insectivorous birds. Thus, a more precise measurement of cumulative insect body size should also include other relevant groups, e.g. Diptera and Coleoptera.

In addition, the use of community-weighted means (CWMs) of body sizes may affect the result: While the unweighted body sizes of species may decrease, changes in abundances may result in increases of the body size CWMs [73]. This is evidenced by the changes in moth and bee cumulative body size with temperature and with total plant biomass, which were opposed to those of the corresponding CWMs. Thus, increases or decreases in average body size can be counterbalanced by changes in overall abundance of large-bodied and small-bodied species. This could be another reason for the positive relationship between insectivorous bird body size and temperature, meaning that at higher elevations, small species may have more individuals than large ones, as compared to lower elevations. In addition, body size CWMs do not account for the dispersion of body sizes in habitats. Dispersion, rather than means, may likely respond to increasing structural complexity of habitats or increasing discontinuity of resource availability across scales [74–75].

Body size distributions probably also depend on other factors not considered in our study [10]. Large body size may be caused by sexual selection or elevated fecundity of large individuals [76]. The interplay of sexual selection for larger individuals and natural selection for smaller ones can complicate patterns [77]. Blanckenhorn [76] proposed selective forces favoring small body size: Larger individuals need more resources, may be preferred by parasites and predators, and may have reduced fecundity if reproducing late. However, quantifying these parameters in a comparative empirical approach across multiple taxa, or guilds along multiple environmental gradients, is difficult.

We used proxies of plant traits to indicate resource availability as a predictor of body size. This was necessary because logistic constraints did not allow for quantifying true nectar availability, or insectivorous bird food supply, among others. The selection of proxies was not arbitrary; higher proportions of insect-pollinated plants should, on average, yield more nectar than lower ones. Higher cumulative body sizes of moths and bees should indicate higher availability of other prey insects eaten by birds. These relationships, while plausible, may not always hold: In certain habitats, chemically or physically well-defended insects could have a larger cumulative body size than the insects from other localities without increasing resource availability for birds. Abundant insect-pollinated plant species with heavy allocation to vegetative compared to reproductive biomass could provide less nectar than rare insect-pollinated species with stronger allocation to reproductive biomass. Eventually, this may lead to a divergence between the proxies and the actual traits measured. While this constraint needs to be considered, the test of hypotheses with proxies may lead to new insights, if the relationships of proxies and true variables hold. This has been seen in the use of "soft" instead of "hard" traits to predict plant photosynthesis and carbon allocation [78], or the common use of precipitation instead of soil water availability to predict vegetation patterns [79].

In summary, major effects of all abiotic predictors on plant functional traits could be confirmed. Temperature affected animal body sizes, albeit in different directions. The more-food hypothesis was, however, hardly supported by our data. If this hypothesis were correct, we should have found some coupling between leaf traits indicating palatability and body sizes of animals, particularly herbivorous guilds. There are several possible explanations. Firstly, the traits describing resource availability may not have been good enough proxies to reveal the true relationships. Secondly, using the community-weighted means (CWMs) of traits and body sizes may have lumped together many species that do not necessarily depend on the same resources. Moth caterpillars are known to be host-specific [70], and the relationship of plants and pollinators is characterized by a large variety of interactions from co-dependent plant-animal species pairs to generalist species [80]. Also, frugivorous and insectivorous birds are likely to be specialized to some degree, making the use of CWMs inappropriate.

The concept behind CWMs is that environmental conditions exert strong selective forces on a trait and filter only a small subset of the total trait expressions of the geographical species pool, leading to convergence of the trait values around a mean describing this subset [81–82]. The large environmental variation along the slopes of Mt. Kilimanjaro, resulting in habitats as diverse as alpine vegetation, rainforests, savanna and fields favored the filtering of plant traits as evidenced by our structural equation model. CWMs have become increasingly popular in plant functional trait studies, mainly because trait means per plot can be directly linked to environmental variables characterizing the plot, thus making statistical analyses relatively easy. Plant-animal interactions, however, seem to be too species-specific to allow the use of community-weighted means, both in plants and animals. For future work, it appears more promising to study pairwise matching of plant and animal traits in ecological networks [83–85] and to correlate these matches with environmental conditions.

## Conclusion

We investigated the response of plant traits to the environment and their effect on the community-weighted mean body size of moths, bees, frugivorous birds, and insectivorous birds at Mount Kilimanjaro, Tanzania. Plant functional traits were selected to reflect resource availability for animals. Structural equation modeling confirmed the response of plant functional traits to the abiotic environment. However, not plant functional traits, but temperature and total plant biomass were the main drivers of mean body size in our study system. Temperature—body size relationships were not always in line with expectations, indicating multiple factors shape the body size distribution of the animal groups investigated. We found no support for the resource availability hypothesis. Possible reasons may be that (i) the plant traits used may not quantify food sources to the desirable detail and (ii) community-weighted means may not be suited to revealing plant-animal interactions sufficiently.

## Supporting information

S1 Table Mean temperature, annual precipitation and disturbance the vegetation types at Mount Kilimanjaro. The sixty plots from twelve vegetation types investigated in this study represent a large part of the habitats present at the Southern slopes of Mount Kilimanjaro, Tanzania. Disturbance was calculated including various aspects of anthropogenic changes to the environment described in S1 File.
(TXT)

S2 Table Pearson correlations between plant functional traits and leaf economics. Leaf economics is the first axis of a PCA including specific leaf area (SLA), leaf dry matter content (LDMC), stem specific density (SSD), leaf nitrogen per unit mass (leaf N_mass_), and leaf phosphorus per unit mass (leaf P_mass_).
(PDF)

S3 Table Plant functional traits, animal body size CWMs, bee cumulative intertegular distance (ITD), and moth cumulative body length. Community-weighted means are given for each of the sixty plots. Bee ITD and bird mass are highly correlated with body size, and have been referred as such in the text. Variables were scaled prior to analysis. NAs indicate no respective species were found in these plots.
(TXT)

S4 Table Coefficients, P-values, generalized R^2^ values, and model probability of the structural equation models. The relationships between the abiotic environment (precipitation, disturbance), total plant biomass, and leaf economics were the same for all models and coefficients and P-values are given only once. All variables were standardized prior to analyses. Unstandardized coefficients were obtained by multiplying standardized coefficients with the ratio of the standard deviations of response and predictor. For each taxonomic group or guild, data is presented for initial models ("hypothesis") and improved models dropping non-significant paths ("significant "). Stars indicate P-values smaller than 0.05, 0.01 and 0.001, respectively.
(PDF)

S1 File Disturbance index calculation. (PDF)
