# Supplementary material for: Relationships between abiotic environment, plant functional traits, and animal body size at Mount Kilimanjaro, Tanzania
Source: PLoS One. 2017 Mar 20;12(3):e0174157. doi: 10.1371/journal.pone.0174157 (PMC5358856; doi:10.1371/journal.pone.0174157)
Supplement: S2 Table — Leaf economics is the first axis of a PCA including specific leaf area (SLA), leaf dry matter content (LDMC), stem specific density (SSD), leaf nitrogen per unit mass (leaf Nmass), and leaf phosphorus per unit mass (leaf Pmass). (PDF) [file pone.0174157.s002.pdf]

Relationships between abiotic environment, plant functional traits, and animal body size at Mount Kilimanjaro, Tanzania

Authors: David Schellenberger Costa*, Alice Classen, Stefan Ferger, Maria Helbig-Bonitz, Marcell Peters, Katrin Böhning-Gaese, Ingolf Steffan-Dewenter, Michael Kleyer

*Corresponding author: david.schellenberger.costa@uni-oldenburg.de

S2_Table. Pearson correlations between plant functional traits and leaf economics.

Leaf economics is the first axis of a PCA including specific leaf area (SLA), leaf dry matter content (LDMC), stem specific density (SSD), leaf nitrogen per unit mass (leaf N_{mass}), and leaf phosphorus per unit mass (leaf P_{mass}).

	LDMC	SSD	Leaf N_{mass}	Leaf P_{mass}	Leaf economics
SLA	-0.72	-0.85	0.77	0.73	0.92
LDMC		0.82	-0.91	-0.62	-0.92
SSD			-0.76	-0.62	-0.92
Leaf N_{mass}				0.56	0.91
Leaf P_{mass}					0.78
