# Supplementary material for: Relationships between abiotic environment, plant functional traits, and animal body size at Mount Kilimanjaro, Tanzania
Source: PLoS One. 2017 Mar 20;12(3):e0174157. doi: 10.1371/journal.pone.0174157 (PMC5358856; doi:10.1371/journal.pone.0174157)
Supplement: S4 Table — The relationships between the abiotic environment (precipitation, disturbance), total plant biomass, and leaf economics were the same for all models and coefficients and P-values are given only once. All variables were standardized prior to analyses. Unstandardized coefficients were obtained by multiplying standardized coefficients with the ratio of the standard deviations of response and predictor. For each taxonomic group or guild, data is presented for initial models ("hypothesis") and improved models dropping non-significant paths ("significant "). Stars indicate P-values smaller than 0.05, 0.01 and 0.001, respectively. (PDF) [file pone.0174157.s004.pdf]

Relationships between abiotic environment, plant functional traits, and animal body size at Mount Kilimanjaro, Tanzania

Authors: David Schellenberger Costa*, Alice Classen, Stefan Ferger, Maria Helbig-Bonitz, Marcell Peters, Katrin Böhning-Gaese, Ingolf Steffan-Dewenter, Michael Kleyer

*Corresponding author: david.schellenberger.costa@uni-oldenburg.de

S4 Table. Coefficients, P-values, generalized R^2 values, and model probability of the structural equation models. The relationships between the abiotic environment (precipitation, disturbance), total plant biomass, and leaf economics were the same for all models and coefficients and P-values are given only once. All variables were standardized prior to analyses. Unstandardized coefficients were obtained by multiplying standardized coefficients with the ratio of the standard deviations of response and predictor. For each taxonomic group or guild, data is presented for initial models ("hypothesis") and improved models dropping non-significant paths ("significant "). Stars indicate P-values smaller than 0.05, 0.01 and 0.001, respectively.

Model	Response	Predictor	Standardized Coefficient	Unstandardized Coefficient	P-value	Marginal R^2	Conditional R^2	Model probability
All	Precipitation					0	0.73	
	Temperature					0	0.96	
	Disturbance	(Intercept)	-0.15	-4.76E-02		0.38	0.91	
	Disturbance	Temperature	0.49	2.40E-02	3.54E-04 ***			
	Leaf economics	(Intercept)	-0.09	-1.84E-01		0.52	0.77	
	Leaf economics	Disturbance	0.43	2.76E+00	0.01 **			
	Leaf economics	Precipitation	0.43	1.25E-03	2.85E-06 ***			
	Total plant biomass	(Intercept)	0.00	0.00		0.32	0.72	
	Total plant biomass	Disturbance	-0.30	-1.54E+02	0.04 *			
	Total plant biomass	Precipitation	0.37	8.73E-02	1.00E-03 **			
Moths hypothesis	Insect-pollinated plants CWM	(Intercept)	0.05	1.37E-02		0.47	0.71	0.55
	Insect-pollinated plants CWM	Leaf economics	0.62	9.02E-02	9.16E-06 ***			
	Insect-pollinated plants CWM	Total plant biomass	0.24	4.30E-04	0.04 *			
	Moth CWM	(Intercept)	0.11	3.31E-01		0.49	0.51	
	Moth CWM	Insect-pollinated plants CWM	-0.08	-8.32E-01	0.61			
	Moth CWM	Leaf economics	-0.15	-2.40E-01	0.35			
	Moth CWM	Temperature	-0.59	-2.87E-01	2.00E-04 ***			
	Moth CWM	Total plant biomass	0.11	2.11E-03	0.39			
Moths significant	Insect-pollinated plants CWM	(Intercept)	0.05	1.37E-02		0.47	0.71	0.76
	Insect-pollinated plants CWM	Leaf economics	0.62	9.02E-02	9.16E-06 ***			
	Insect-pollinated plants CWM	Total plant biomass	0.24	4.30E-04	0.04 *			
	Moth CWM	(Intercept)	0.14	4.53E-01		0.49	0.51	
	Moth CWM	Temperature	-0.76	-3.70E-01	2.52E-06 ***			
Bees hypothesis	Bee CWM	(Intercept)	0.02	1.10E-01		0.53	0.53	0.27
	Bee CWM	Disturbance	0.40	6.56E+00	0.03 *			
	Bee CWM	Insect-pollinated plants CWM	-0.11	-1.97E+00	0.37			
	Bee CWM	Temperature	0.38	3.00E-01	0.02 *			
	Bee CWM	Total plant biomass	0.52	1.63E-02	4.42E-04 ***			

	Insect-pollinated plants CWM	(Intercept)	0.05	1.37E-02		0.47	0.71	
	Insect-pollinated plants CWM	Leaf economics	0.62	9.02E-02	9.16E-06 ***			
	Insect-pollinated plants CWM	Total plant biomass	0.24	4.30E-04	0.04 *			
Bees significant	Bee CWM	(Intercept)	0.01	7.50E-02		0.58	0.66	0.41
	Bee CWM	Disturbance	0.32	5.27E+00	0.04 *			
	Bee CWM	Temperature	0.49	3.88E-01	1.10E-03 **			
	Bee CWM	Total plant biomass	0.50	1.57E-02	0.01 *			
	Insect-pollinated plants CWM	(Intercept)	0.05	1.37E-02		0.47	0.71	
	Insect-pollinated plants CWM	Leaf economics	0.62	9.02E-02	9.16E-06 ***			
	Insect-pollinated plants CWM	Total plant biomass	0.24	4.30E-04	0.04 *			
Frugivorous birds hypothesis	Bird-dispersed fruit CWM	(Intercept)	0.01	2.37E+03		0.17	0.89	0.36
	Bird-dispersed fruit CWM	Leaf economics	-0.26	-4.29E+04	0.34			
	Bird-dispersed fruit CWM	Total plant biomass	0.14	2.83E+02	0.05 *			
	Frugivorous bird CWM	(Intercept)	0.03	2.95E+00		0.35	0.41	
	Frugivorous bird CWM	Bird-dispersed fruit CWM	0.49	1.42E-04	0.18			
	Frugivorous bird CWM	Temperature	-0.21	-3.18E+00	0.54			
	Frugivorous bird CWM	Total plant biomass	-0.05	-3.27E-02	0.82			
Frugivorous birds significant	Bird-dispersed fruit CWM	(Intercept)	0.01	2.37E+03		0.17	0.89	0.7
	Bird-dispersed fruit CWM	Leaf economics	-0.26	-4.29E+04	0.34			
	Bird-dispersed fruit CWM	Total plant biomass	0.14	2.83E+02	0.05 *			
	Frugivorous bird CWM	(Intercept)	0.12	1.18E+01		0.16	0.16	
	Frugivorous bird CWM	Temperature	-0.44	-6.64E+00	0.05 *			
Insectivorous birds hypothesis	Insect-pollinated plants CWM	(Intercept)	0.05	1.37E-02		0.47	0.71	0.25
	Insect-pollinated plants CWM	Leaf economics	0.62	9.02E-02	9.16E-06 ***			
	Insect-pollinated plants CWM	Total plant biomass	0.24	4.30E-04	0.04 *			
	Insectivorous bird CWM	(Intercept)	-0.01	-1.01E-01		0.33	0.33	
	Insectivorous bird CWM	Temperature	0.46	7.65E-01	9.41E-04 ***			
	Insectivorous bird CWM	Total bee biomass	0.01	4.48E-05	0.96			
	Insectivorous bird CWM	Total moth biomass	-0.02	-5.49E-04	0.85			
	Insectivorous bird CWM	Total plant biomass	-0.26	-1.70E-02	0.05 *			
	Total bee biomass	(Intercept)	0.00	0.00		0.31	0.31	
	Total bee biomass	Disturbance	-0.17	-9.20E+02	0.39			
	Total bee biomass	Insect-pollinated plants CWM	0.06	3.56E+02	0.61			
	Total bee biomass	Temperature	-0.14	-3.68E+01	0.41			
	Total bee biomass	Total plant biomass	-0.62	-6.34E+00	3.06E-05 ***			
	Total moth biomass	(Intercept)	0.00	0.00		0.27	0.27	
	Total moth biomass	Insect-pollinated plants CWM	0.22	3.40E+02	0.16			
	Total moth biomass	Leaf economics	-0.37	-8.56E+01	0.04 *			

	Total moth biomass	Temperature	0.65	4.58E+01	4.06E-05 ***			
	Total moth biomass	Total plant biomass	0.01	4.21E-02	0.90			
Insectivorous birds significant	Insect-pollinated plants CWM	(Intercept)	0.05	1.37E-02		0.47	0.71	0.81
	Insect-pollinated plants CWM	Leaf economics	0.62	9.02E-02	9.16E-06 ***			
	Insect-pollinated plants CWM	Total plant biomass	0.24	4.30E-04	0.04 *			
	Insectivorous bird CWM	(Intercept)	-0.01	-1.03E-01		0.33	0.33	
	Insectivorous bird CWM	Temperature	0.45	7.45E-01	3.07E-04 ***			
	Insectivorous bird CWM	Total plant biomass	-0.26	-1.73E-02	0.02 *			
	Total bee biomass	(Intercept)	-0.05	-8.72E+01		0.23	0.3	
	Total bee biomass	Total plant biomass	-0.45	-4.64E+00	8.75E-04 ***			
	Total moth biomass	(Intercept)	-0.01	-5.08E+00		0.22	0.24	
	Total moth biomass	Temperature	0.44	3.12E+01	2.49E-03 **			
