# Supplementary material for: Relationships between abiotic environment, plant functional traits, and animal body size at Mount Kilimanjaro, Tanzania
Source: PLoS One. 2017 Mar 20;12(3):e0174157. doi: 10.1371/journal.pone.0174157 (PMC5358856; doi:10.1371/journal.pone.0174157)
Supplement: S1 File — (PDF) [file pone.0174157.s005.pdf]

Relationships between abiotic environment, plant functional traits, and animal body size at Mount Kilimanjaro, Tanzania

Authors: David Schellenberger Costa*, Alice Classen, Stefan Ferger, Maria Helbig-Bonitz, Marcell Peters, Katrin Böhning-Gaese, Ingolf Steffan-Dewenter, Michael Kleyer
*Corresponding author: david.schellenberger.costa@uni-oldenburg.de

S1_File. Disturbance index calculation.

Disturbance is a broad term including many distinct and sometimes habitat-specific alterations in ecosystems [1]. By recording a large range of possible perturbations in our plots, we derived a meaningful classification of the degree of disturbance. Our index was calculated as a composite metric including the effects of biomass removal, input of chemicals and landscape composition in the vicinity of the research plots (1500 m radius). Biomass removal has a strong impact on vegetation structure and biodiversity by impeding slow-growing species from reproducing [2]. Chemicals strongly alter soil nutrient availability in the case of fertilizers and kill weeds and natural enemies as fungi or insects. A strongly disturbed landscape matrix can influence plants and animals through edge effects [3]. Historic timber harvest (past 50 years) and fire events were retrieved from long-term data and historic accounts. Current timber harvest, mowing, grazing, ploughing, and input of chemicals were recorded on site in natural habitats and through interviews with land owners or managers in managed areas. The fraction of managed habitats in the research plot vicinity was based on a maximum likelihood land-cover classification applied to four almost cloud-free visible-light Terra-ASTER scenes covering the time of data collection (https://asterweb.jpl.nasa.gov/index.asp). See [4] for similar composite indices in temperate landscapes. Raw value ranges are given in parentheses below the variables. However, variables were linearly standardized to [0,1] before calculating sums.

Biomass removal (Numeric scale, unit of all variables: Removed fraction of resource per year):

$$biomass\ removal = \frac{current\ timber\ harvest}{(0-0.083)} + \frac{historic\ timber\ harvest}{(0-0.03)} + \frac{mowing}{(0-1)} + \frac{grazing}{(0-0.05)} + \frac{ploughing}{(0-1)} + \frac{fire\ (last\ 30\ yrs)}{(0-0.05)}$$

Input of chemicals (Ordinal scale: 0 = no/very low, 1 = intermediate, 2 = high):

$$input\ of\ chemicals = \frac{pesticide\ application}{(0-2)} + \frac{fungicide\ application}{(0-2)} + \frac{herbicide\ application}{(0-2)} + \frac{fertilizer}{(0-2)} + \frac{irrigation}{(0-1)}$$

Landscape composition (Numeric scale, unitless):

$$landscape\ composition = \frac{fraction\ of\ managed\ habitats\ in\ plot\ vicinity\ (radius\ 1500\ m)}{(0\ -1)}$$

Summary formula (Numeric scale, unitless):

$$disturbance = \frac{biomass\ removal}{(0-2.71)} + \frac{input\ of\ chemicals}{(0-5)} + \frac{landscape\ composition}{(0-1)}$$

1. White PS, Jentsch A. The search for generality in studies of disturbance and ecosystem dynamics. Progress in botany. Berlin, Heidelberg: Springer; 2001. p. 399-450.
2. Lambers H, Chapin FS, Pons T. Plant Physiological Ecology. New York: Springer; 2008.
3. Murcia C. Edge effects in fragmented forests: implications for conservation. Trends in Ecology & Evolution. 1995;10(2):58-62.
4. Kleyer M. Distribution of plant functional types along gradients of disturbance intensity and resource supply in an agricultural landscape. Journal of Vegetation Science. 1999;10(5):697-708.
